# Evaluation of reproductive and renal toxicity of varenicline in male rats

**DOI:** 10.22038/IJBMS.2019.13986

**Published:** 2019-12

**Authors:** Fatih Oguz, Ali Beytur, Elif Taslidere, Hakan Parlakpinar, Hilal Kurnaz Oguz, Alaaddin Polat, İbrahim Topcu, Nigar Vardi, Engin Burak Selcuk

**Affiliations:** 1 Inonu University, School of Medicine, Department of Urology, Malatya, Turkey; 2 Inonu University, School of Medicine, Department of Histology and Embryology, Malatya, Turkey; 3 Inonu University, School of Medicine, Department of Pharmacology, Malatya, Turkey; 4 Malatya State Hospital, Department of Internal Medicine, Malatya, Turkey; 5 Inonu University, School of Medicine, Department of Physiology, Malatya, Turkey; 6 Inonu University, School of Medicine, Department of Family Medicine, Malatya, Turkey

## Abstract

**Objective(s):**

Varenicline is a selective partial agonist for the nicotinic acetylcholine receptor a4b2 subtype, which is widely used to treat smoking addiction. However, there is still no data about its potential toxic effects on tissues. In this study, we aimed to determine the varenicline-induced toxicity on reproductive and renal tissues in rats.

**Materials and Methods:**

Rats were randomly divided into two groups: control (n=10) and varenicline (n=24). Then, rats in each group were sub-divided equally as acute and chronic groups. The control rats were orally given distilled water only. Varenicline was administrated orally as follows: 1^st^–3^rd^ days 9 µg/kg/day, 4^th^–7^th^ days 9 µg/kg twice daily, and 8^th^–90^th^ days 18 µg/kg twice daily. The rats of acute and chronic groups were sacrificed on the 45^th^ and 90^th^ days, respectively. Some tissue markers related to oxidative stress were measured, and sperm characteristics were observed.

**Results:**

In the acute group, varenicline led to a significant decrease in SOD activities in both kidney and testis tissues. In the chronic group, varenicline significantly increased MDA and MPO production, and reduced CAT and GPx levels in the kidneys and testes. Also, SOD and GSH levels significantly decreased in the testes. Moreover, sperm characteristics were negatively affected; histopathological deformation was observed in the testes and kidneys in all groups.

**Conclusion:**

This study showed that varenicline could detrimentally affect the kidneys and testes in both acute and chronic term usage. Further studies will provide more insights into the molecular dynamics of this damage.

## Introduction

Smoking tobacco is regarded as a significant contributing factor to early death and is associated with severe health problems including myocardial infarction, stroke, chronic obstructive pulmonary disease, and cancer. Thus, the cessation of smoking is a crucial medical challenge. In North America, the food and drug administration (FDA) has approved three first-line pharmacotherapies for smoking cessation: Nicotine replacement therapy (NRT), varenicline, and sustained-release bupropion (1). 

Varenicline is a highly selective partial agonist for the nicotinic acetylcholine receptor a4b2 subtype, which is assumed to be responsible for mediating the nicotine addiction. Varenicline also acts as a partial agonist to relieve nicotine craving and withdrawal, but an antagonist reducing the psychogenic reward associated with smoking (2). Randomized clinical trials have demonstrated that varenicline increases the chance of successful long-term smoking cessation by 2-3 fold when compared with smoking cessation attempts without any pharmacological assistance (3).

The most commonly reported adverse effects of varenicline are vomiting and flatulence observed in more than 5% of patients, and the incidence of acute renal failure is less than 1% (4). Anxiety, depression, aggression, disorientation, and decreased libido are other infrequent side effects observed in randomized clinical studies (2, 4). A meta-analysis indicated an increased risk of severe adverse cardiovascular effects associated with varenicline (5). 

Urologists are frequently being confronted with the question regarding whether the use of varenicline has any detrimental impact on the male urinary and reproductive systems such as sperm characteristics and infertility. To date, no studies have investigated the effects of varenicline on renal function and the reproductive system. The present study aimed to determine the acute and chronic effects of varenicline on kidney tissue and the reproductive system in male rats. This study included the examination of histological features and oxidative stress in the kidney and testicular tissues, sperm characteristics, and reproductive organ weight.

## Materials and Methods

***Animals***

This experimental study was designed according to Animal Research: Reporting of *In Vivo* Experiments (ARRIVE) Guidelines (6). A total of 34 male Wistar albino rats (aged 10–12 weeks; weight, 250–300 g, Inonu University, Experimental Animal Research and Application Center) were housed in an air-conditioned room with a 12 hr light-dark cycle, constant temperature (22±2 ^°^C), and relative humidity (65–70%). The rats were provided standard commercial pellets and water *ad libitum.* All experimental protocols were approved by Animal Care and Use Committee of Inonu University School of Medicine (Malatya, Turkey).

***Experimental Protocol***

The rats were randomly divided into two groups: the control (C; n=10) and varenicline groups (V; n=24). Then, the rats in each group were sub-divided equally into acute (C1 or V1) and chronic (C2 or V2) groups. The control rats were treated with distilled water through oral gavage only. The doses and treatment duration of varenicline (Champix 1 mg tablet^®^, Pfizer Corp., Istanbul, Turkey.) were selected according to the varenicline treatment scheme for humans based on a modified conversion table of animal doses to human-equivalent doses (7). Varenicline was administrated orally as follows: days 1–3, 9 µg/kg/day; days 4–7, 9 µg/kg twice daily; and days 8–90 (total, 83 days), 18 µg/kg twice daily. On day 45, the rats in the acute groups [C1 (n=5) and V1 (n=12)] were sacrificed and on day 90, the rats in the chronic groups [C2 (n=5) and V2 (n=12)] were sacrificed with a combination of anesthetics: 80 mg/kg ketamine and 5 mg/kg xylazine. Then kidney and testis tissues were quickly removed for biochemical and histopathological examinations. 

***Biochemical analysis***

The malondialdehyde (MDA) content of the homogenates was determined spectrophotometrically by measuring the presence of thiobarbituric acid reactive substances (TBARS) (8). The absorbance at 532 nm was measured by a spectrophotometer (catalog number: UV-1601; supplier: Shimadzu, Kyoto, Japan). The results were expressed in pmol/mg protein according to a standard graph, which was prepared from measurements of standard solutions (1,1,3,3-tetramethoxypropane).

The glutathione (GSH) content in the kidney homogenate was measured at 412 nm using the method of Sedlak and Lindsay (9). The GSH level was expressed in nmol/ml. 

Superoxide dismutase (SOD) activity was measured by the inhibition of nitroblue tetrazolium (NBT) reduction due to O_2_ generated by the xanthine/xanthine oxidase system (10). One unit of SOD activity was defined as the amount of protein causing 50% inhibition of the NBT reduction rate. The product was spectrophotometrically evaluated at 560 nm. The results are expressed in U/mg protein.

Catalase (CAT) activity was determined according to the method of Aebi (11). The enzymatic decomposition of H_2_O_2_ was directly detected by measuring the decrease in absorbance at 240 nm. The difference in absorbance per time unit was used as a measure of CAT activity. The enzyme activities are stated in kU/mg protein.

GSH peroxidase (GPx) activity was measured via the method of Paglia and Valentine (12). The decrease in absorbance at 340 nm was measured; GPx activity was expressed as IU/mg protein (12)

Myeloperoxidase (MPO) activity was determined by using a 4-aminoantipyrine/phenol solution as the substrate for MPO-mediated oxidation by H_2_O_2_ and measuring the change in the absorbance at 510 nm (13). One unit of MPO activity was defined as the amount causing the degradation of 1 µmol H_2_O_2_/min at 25 ^°^C. The results were provided in U/g protein.

Furthermore, trunk blood from the heart was extracted to evaluate serum levels of blood urea nitrogen (BUN), creatinine (Cr), and testosterone using an Olympus Autoanalyzer (Olympus Instruments, Tokyo, Japan).

***Histopathological analysis***

Paraffin-embedded blocks of kidney tissue were sectioned at 5 µm thickness and stained with hematoxylin-eosin (H&E) and periodic acid-Schiff (PAS) stain at 5 min at room temperature (18–26 °C). At a magnification of x40; 20 randomly selected glomeruli in the cortex of section were evaluated for each section. For each glomerulus, two different diameters (vertical and horizontal) of each glomerulus were measured, and the average diameter was calculated.

Sections of testis tissue were stained with H&E. For each slice, 100 tubules were classified as being intact, sloughing, atrophic, or degenerated. Seminiferous tubule diameters and germinative cell layer thickness were measured using the Leica Q Win Plus Image Analysis System (Leica Microsystems, Cambridge, U.K.) at a magnification of x20. 

For immunohistochemical analysis, thick sections of kidney and testis tissues were mounted on polylysine-coated slides. After rehydrating, the samples were immersed in citrate buffer (pH 7.6) and boiled by heating in a microwave oven for 20 min. After cooling for 20 min at room temperature, the sections were washed with phosphate-buffered saline (PBS). Subsequently, the sections were incubated in 0.3% H_2_O_2_ for 7 min and then washed with PBS. Sections were incubated with primary rabbit-monoclonal antibody to cysteine aspartate-specific proteinase (caspase)-3 (Thermo Fisher Scientific, Inc., Waltham, MA, USA) antibody for 30 min, rinsed in PBS, incubated with biotinylated goat anti-polyvalent for 10 min, and streptavidin-peroxidase for 10 min at room temperature. The staining procedure was completed by incubation with chromogen+substrate for 15 min, and slides were counterstained with Mayer’s hematoxylin for 1 min at room temperature, rinsed with tap water, and dehydrated. Seminiferous tubules and cortical renal tubules with staining for caspase-3 were counted using a Leica Q Win Image Analysis System (Version V2.6). For each specimen, 100 tubules were examined at a magnification of x20.

***Evaluation of sperm parameters***

The epididymal sperm concentration was determined with a hemocytometer using a modified method described in the literature (14) after the supernatant fluids containing all epididymal sperm cells were counted using a light microscope at a magnification of x20. To determine sperm vitality, 40 μl of freshly liquefied semen was thoroughly mixed with 10 μl of eosin Y (1℅), and one drop of this mixture was transferred to a clean slide. A total of one hundred sperms were counted in each slide at a magnification of x100 under oil immersion. Sperms that were stained pink or red were regarded as dead and unstained sperms were considered as viable.

***Statistical analysis***

The sample sizes required for a statistical power of 0.80 were estimated using the NCSS software. Data were analyzed using the SPSS software program for Windows, version 18.0 (SPSS Inc, Chicago, IL, USA). The normality of the distribution was confirmed using the Kolmogorov–Smirnov test. According to the results obtained from the normality test, the Kruskal–Wallis H test was used for the statistical analysis, as appropriate. After significance was indicated by the Kruskal–Wallis H test, a Conover test (*a post-hoc* *test*) was also performed for biochemical and histopathological results. *P-value*<0.05 was considered to indicate a statistically signiﬁcant difference. The values are expressed as the median (range) unless otherwise specified.

## Results

***Organ weight and biochemical results***

Testis and kidney weights with oxidant/antioxidant parameters (MDA, SOD, CAT, GPx, GSH, and MPO) were presented in Tables 1-3. During the acute treatment phase, varenicline led to a significant decrease in the left renal weight and SOD activities in the kidney and testis tissue when compared with those in the control group. Furthermore, an increase in MDA levels and MPO activities and a decrease in CAT and GPx activities were observed; however, the changes did not reach significant levels. On the other hand, in the chronic treatment group, varenicline significantly increased MDA and MPO production while it reduced CAT and GPx levels in the kidney and testis tissues. In addition, the activities of SOD and GSH decreased significantly only in the testis tissue. Significant differences in the MDA, MPO, and GSH levels were observed between the acute and chronic varenicline treatment groups. In particular, in the kidney and testis tissues in the chronic group, MDA and MPO levels were significantly higher, and GSH levels were significantly lower than those in the acute group. Acute and chronic varenicline treatment caused significant decreases in the right and left epididymis weights compared with those in the control groups (*P*<0.05). Similarly, chronic varenicline treatment led to significant reductions in the right and left epididymis weights when compared with those in the acute varenicline group (*P*<0.05).

No differences in BUN, Cr, and testosterone levels were observed between the acute and chronic varenicline treatment groups (Table 4).

***Histopathological results***

*Kidney tissue observations*

**Figure 1:**
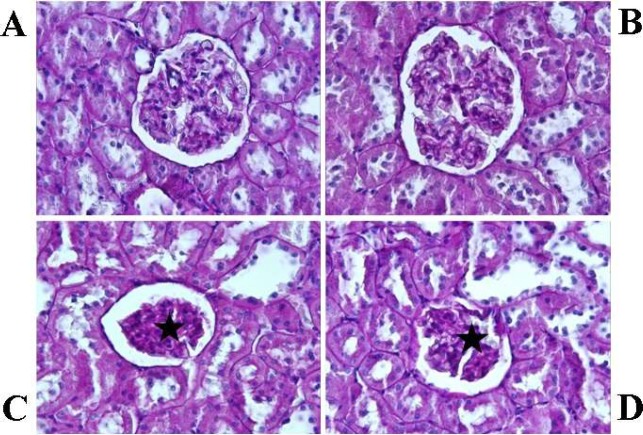
Histological analysis of kidney tissues (periodic acid Schiff staining; magnification, x40). Acute control group (A) and chronic control group (B), the histological appearance of glomeruli is intact. Acute varenicline group (C) and chronic varenicline group (D), the glomerular diameter is significantly decreased in comparison to the control groups (stars)

**Figure 2:**
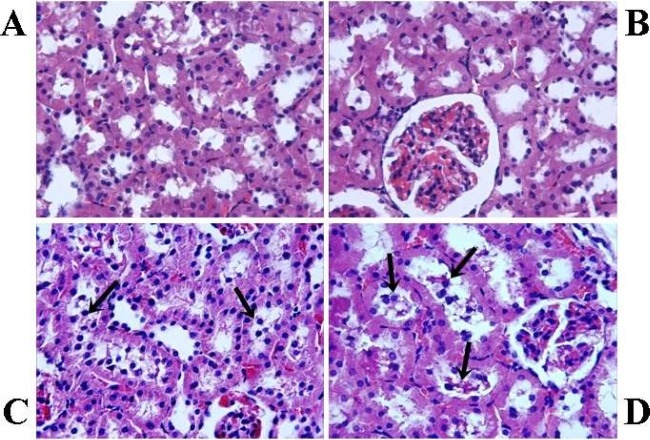
Histological analysis of kidney tissues (H&E staining; magnification, x40). Acute control group (A) and chronic control group (B), the histological appearance of the renal tubule is normal. Acute varenicline group (C) and chronic varenicline group (D), in the group V desquamated cells are more frequent in the lumina of tubules (arrows)

The histological appearance of glomeruli was intact in C1 and C2 groups (Figures 1A and 1B). However, application of varenicline caused injury to the glomeruli. The most remarkable histological damage was glomerular shrinkage in V1 and V2 groups (Figures 1C and 1D). The diameter of the glomeruli was measured as 72.12±8.18 (mean±SD) and 70.49±8.15 (mean±SD) in V1 and V2 groups, respectively. However, the mean diameter of the glomeruli was lower in V1 and V2 groups compared with those in the respective control group (*P*<0.05). The control groups exhibited normal histological renal tubules (Figures 2A and 2B). On the other hand, some of the cortical tubules in V1 and V2 groups showed morphological changes such as desquamation (Figures 2C and 2D). The mean diameters of glomeruli are presented in Table 5. 

*Caspase-3 activity assay*

**Figure 3:**
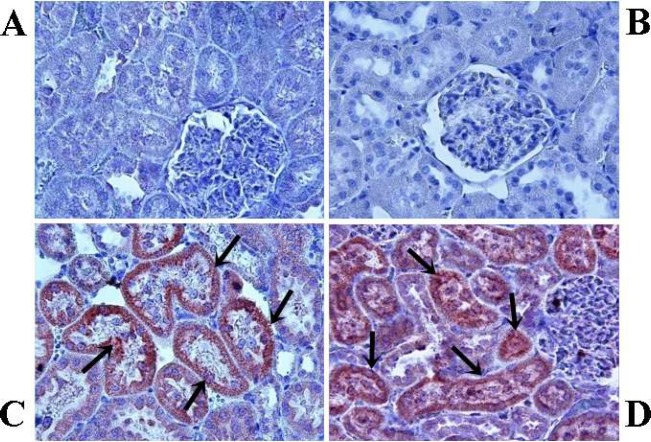
Immunohistochemical staining for active caspase-3 (brown staining; magnification, x20). Acute control group (A) and chronic control group (B), no caspase-3-positive tubules are present; acute varenicline group (C) and chronic varenicline group (D), numerous caspase3+cells are observed (arrows)

In the control groups, the cortical renal tubules did not exhibit any staining for caspase-3 (Figures 3A and B). However, in V1 and V2 groups, caspase-3 positive tubules were found to be significantly increased when compared with the control groups (*P*<0.05). (Figures 3C and 3D). Also, caspase-3 activity was significantly increased in the V2 group when compared with the V1 group. The percentage of tubules stained with caspase-3 are shown in Table 5.

*Testis tissue observations*

**Figure 4:**
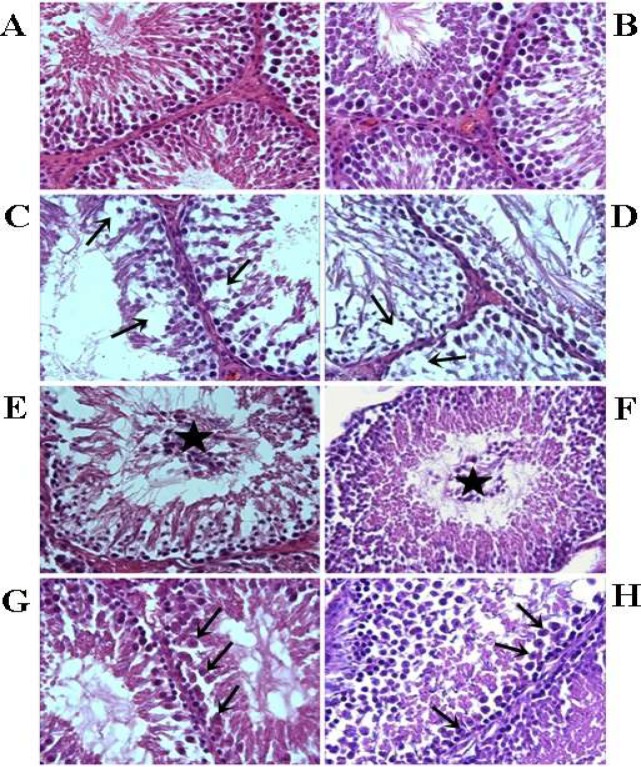
Acute control group (A) and chronic control group (B), the seminiferous epithelium in the testis is intact and exhibits a normal association of germ cells. Acute varenicline group (C) and chronic varenicline group (D), atrophic tubules and loss of the germ cells are evident (arrows). Acute varenicline group (E) and chronic varenicline group (F), sloughing tubules and the shedding of germ cells into the tubular lumina (stars). Furthermore, acute varenicline group (G) and chronic varenicline group (H), degeneration of tubules and arrested spermatocytes in various stages of division (arrows) were present. (H&E staining; magnification, x40)

Germinal cells were organized in concentric layers, and seminiferous tubules in all stages of spermatogenesis were observed in the control groups (Figures 4A and 4B). However, severe degenerative changes in seminiferous tubules were observed in the experimental groups, whereas some of the tubules were atrophic. A significant loss in germ cells was observed in this seminiferous tubules (Figures 4C and 4D). In the spermatogenetic layers, numerous round germ cells were observed in certain seminiferous lumina in this group (Figures 4E and F). In certain other seminiferous tubules, spermatogenic cells arrested at various stages of division were observed (Figures 4G and H). In the V2 group, the number of affected tubules was significantly higher than that in the V1 group (*P*<0.05). Furthermore, the mean seminiferous tubule diameter and germinal epithelium thickness were obviously decreased in V1 and V2 groups compared with those in the control groups. This reduction of tubule diameter and cell layer thickness was more marked in the V2 group compared with the V1 group. Diameters of seminiferous tubules and germinal cell layer thickness are given in Table 6.

***Results of apoptotic germ cell assay***

**Figure 5:**
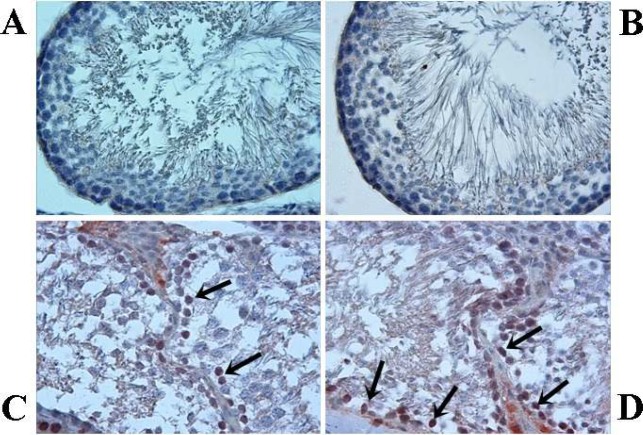
Immunohistochemical staining of testis for active caspase-3. Acute control group (A) and chronic control group (B) exhibit no caspase three immunoreactivity in germ cells. Acute varenicline group (C) and chronic varenicline group (D) exhibit caspase-3 positive germ cells (arrows; magnification, x40)

In the present study, caspase-3 staining revealed no apoptotic cells in the control groups (Figures 5A and 5B). However, in the experimental groups, certain spermatogenic cells exhibited positive staining for caspase-3 (Figures 5C and D). No statistically significant changes in the number of tubules immunostained for caspase-3 were identified between V2 group and V1 groups (*P*>0.05). The numbers of tubules in the testis with staining for caspase-3 are presented in Table 7.

***Assessment of epididymal sperm parameters***

**Figure 6:**
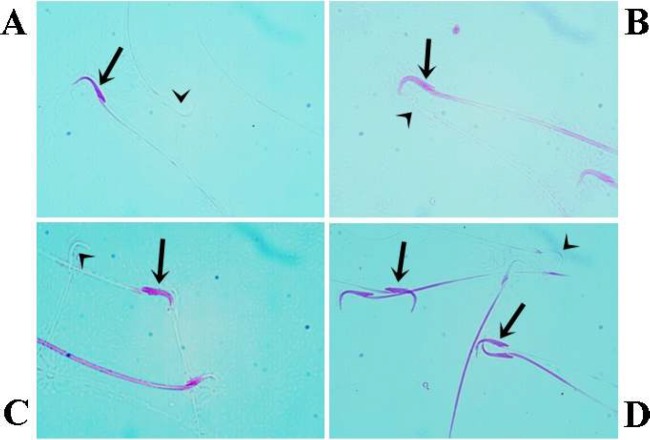
Sperm viability. Acute control group (A), Chronic control group (B), Acute varenicline group (C), chronic varenicline group (D); Dead (pink) and live (nonstained) sperms are indicated by arrows and arrowheads, respectively. The number of dead sperms in the chronic varenicline group was higher than that in the acute varenicline group (eosin staining; magnification, x100)

While the sperms with intact membrane structure were not stained, damaged plasma membranes were stained eosinophilic. The effects of acute and chronic varenicline treatment on epididymal sperm concentration, sperm viability, and abnormal sperm rate were presented in Table 8. The sperm concentration and vitality of the left testis of rats of the experimental groups decreased significantly in comparison with those of groups C1 and C2 (*P*<0.05). (Figures 6A, B, C, and D). Sperm concentration and vitality were lower in the V2 group compared with the V1 group. The sperm concentration and vitality are shown in Table 8.

**Table 1:** Changes in biochemical oxidant and antioxidant parameters in kidney tissues of rats in acute and chronic varenicline as well as control groups

		**MDA** nmol/g tissue	**SOD** U/g protein	**CAT** K/g protein	**GPx** U/mg protein	**GSH** µmol/g tissue	**MPO** U/g protein
**Acute**	Control group	7.05 (5.78-9.08)	33.34 (28.24-37.40)	44.51 (34.69-46.56)	4.42 (3.60-8.14)	0.33 (0.22-0.39)	25.20 (16.72-45.49)
	Varenicline group	7.69 (5.78-9.46)	24.90^a^ (16.51-29.92)	37.96 (21.21-55.06)	4.13 (2.00-6.58)	0.38 (0.30-0.50)	30.07 (19.00-47.98)
**Chronic**	Control group	6.58 (5.44-8.87)	39.78 (33.34-47.40)	44.69 (36.56-62.63)	4.70 (3.80-8.52)	0.31 (0.21-0.36)	16.72 (10.62-25.21)
	Varenicline group	20.59^b,c^ (13.54-29.41)	22.74^b^ (11.14-47.40)	27.46^b^ (17.61-38.36)	3.21^b^ (2.07-5.92)	0.25^c^ (0.22-0.52)	51.40^b,c^ (30.20-67.98)

^a^ significantly different compared with the acute control group (*P≤*0.05), ^b^ significantly different compared with the chronic control group (*P≤*0.05), ^c^ significantly different compared with the acute varenicline group (*P≤*0.05)

MDA, malondialdehyde; SOD, superoxide dismutase; CAT, catalase; GSH, glutathione; GPx, GSH peroxidase; MPO, myeloperoxidase

Values are expressed as the median (range)

**Table 2:** Changes in biochemical oxidant and antioxidant parameters in testis tissues of rats in acute and chronic varenicline as well as control groups

		**MDA** nmol/g tissue	**SOD** U/g protein	**CAT** K/g protein	**GPx** U/mg protein	**GSH** µmol/g tissue	**MPO** U/g protein
**Acute**	Control group	8.53 (7.85-10.00)	51.24 (45.77-57.91)	13.57 (12.90-18.59)	4.40 (2.65-6.78)	0.43 (0.25-0.53)	17.40 (13.10-19.37)
	Varenicline group	8.87 (5.81-12.63)	42.65^a^ (34.49-50.15)	9.61 (5.71-17.12)	5.65 (3.91-8.55)	0.31 (0.29-0.46)	16.24 (11.66-23.98)
**Chronic**	Control group	8.53 (6.85-9.73)	61.24 (57.31-65.77)	17.05 (14.57-18.90)	6.78 (5.76-9.06)	0.53 (0.49-0.65)	13.65 (12.27-15.37)
	Varenicline group	23.42^b,c^ (16.40-29.31)	27.93^b,c^ (15.36-42.43)	5.49^b,c^ (2.92-7.81)	2.75^b,c^ (1.11-4.54)	0.24^b,c^ (0.19-0.32)	30.16^b,c^ (23.96-52.45)

^a^ significantly different compared with the acute control group (*P≤*0.05), ^b^ significantly different compared with the chronic control group (*P≤*0.05), ^c^ significantly different compared with the acute varenicline group (*P≤*0.05)

MDA, malondialdehyde; SOD, superoxide dismutase; CAT, catalase; GSH, glutathione; GPx, GSH peroxidase; MPO, myeloperoxidase

Values are expressed as the median (range)

**Table 3.** Changes in the weights of left and right kidneys, testis and epidiydimal weights of rats in acute and chronic varenicline as well as control groups

		**Weight of right kidney** (g)	**Weight of left kidney** (g)	**Weight of right testis** (g)	**Weight of left testis** (g)	**Weight of right** **epididymis** (g)	**Weight of left** **epididymis** (g)
**Acute**	Control group	1.27 (1.21-1.67)	1.28 (1.19-1.41)	1.36 (1.31-1.51)	1.36 (1.32-1.48)	0.64 (0.62-0.65)	0.63 (0.54-0.71)
	Varenicline group	1.16 (1.11-1.51)	1.09^a^ (1.00-1.37)	1.34 (1.24-1.44)	1.32 (1.02-1.43)	0.57^a^ (0.52-0.62)	0.57^a^ (0.52-0.62)
**Chronic**	Control group	1.48 (1.04-1.61)	1.30 (1.03-1.59)	1.45 (1.19-1.73)	1.48 (1.17-1.77)	0.66 (0.64-0.72)	0.68 (0.55-0.72)
	Varenicline group	1.17 (1.03-1.32)	1.18 (1.06-1.36)	1.36 (1.26-1.59)	1.34 (1.22-1.45)	0.45^b,c^ (0.41-0.56)	0.44^b,c^ (0.39-0.51)

^a^ significantly different compared with the acute control group (*P≤*0.05), ^b^ significantly different compared with the chronic control group (*P≤*0.05), ^c^ significantly different compared with the acute varenicline group (*P≤*0.05)

Values are expressed as the median (range)

**Table 4:** Changes of blood urea nitrogen, creatinine, and testosterone levels in the serum

		**BUN** **mg/dl**	**Cr** **mg/dl**	**Testosterone** **ng/dl**
**Acute**	Control group	18.8±1.79	0.52±0.01	1.62±0.40
	Varenicline group	18.08±1.93	0.50±0.04	0.99±0.47
**Chronic**	Control group	18.80±2.49	0.51±0.04	2.09±0.97
	Varenicline group	17.58±1.62	0.51±0.03	1.79±1.05

BUN: Blood urea nitrogen; Cr: Creatinine

Values are expressed as mean±SD

**Table 5:** Mean diameters of glomeruli and number of tubules stained with caspase-3 in the renal cortex [Median (min-max)]

		**Glomerulus diameter** (µm)	**Caspase-3(+) tubule** (µm)
**Acute**	Control group	91 (77-110)	7 (5-10)
	Varenicline group	72 (50-90)^a^	22 (10-30)^a^
**Chronic**	Control group	94 (75-111)	6 (5-11)
	Varenicline group	70 (51-88)^b,c^	29.58 (20-35)^d, e^

^a^ significant decrease (*P<*0.05), vs acute control group, ^b^ significant decrease (*P<*0.05), vs chronic control group, ^c^ not significant decrease (*P<*0.05), vs acute varenicline group, ^d^ significant increase (*P<*0.05), vs chronic control group, ^e^ significant increase (*P<*0.05), vs acute varenicline group

Values are expressed as the median (range)

**Table 6:** Diameter of seminiferous tubules and germinal cell layer thickness

		**Seminiferous tubule diameter** (µm)	**Thickness of the seminiferous epithelium** (µm)
**Acute**	Control group	292 (222-405)	42.47 (29-59)
	Varenicline group	267 (204-353)^a^	28.18(11-40)^a^
**Chronic**	Control group	281 (213-362)	33.75 (26-43)
	Varenicline group	246 (190-332)^b,c^	27.11(14-37)^b,c^

^a^ significant decrease (*P<*0.05), vs acute control group, ^b^ significant decrease (*P<*0.05), vs chronic control group, ^c^ significant decrease (*P<*0.05), vs acute varenicline group

Values are expressed as the median (range)

**Table 7:** Percentages of histopathologic classification of seminiferous tubules and number of tubules stained with caspase-3 in the testis

		**Intact tubul** (%)	**Sloughing tubul** (%)	**Atrophic tubul** (%)	**Degenerated tubul** (%)	**Caspase-3 (+) tubul** (%)
**Acute**	Control group	90 (85-90)	5 (0-5)	5 (5-10)	0 (0-5)	0 (0-0)
	Varenicline group	32 (30-40)^a^	15 (5-35)^c^	37 (25-50)^c^	12.5 (10-20)^c^	9 (5-15)
**Chronic**	Control group	90 (80-90)	5 (5-10)	5 (5-5)	0 (0-5)	0(0-0)
	Varenicline group	25 (25-35)^b,e^	20 (15-25)^d,f^	40 (30-55)^d,f^	15 (10-20)^d,f^	11(10-20)^g^

^a^ significant decrease (*P<*0.05), vs acute control group, ^b ^significant decrease (*P<*0.05), vs chronic control group, ^c ^significant increase (*P<*0.05), vs acute control group, ^d ^significant increase (*P<*0.05), vs chronic control group, ^e ^significant decrease (*P<*0.05), vs acute varenicline group, ^f ^significant increase (*P<*0.05), vs acute varenicline group, ^g ^not significant change (*P>*0.05) vs acute varenicline group

Values are expressed as the median (range)

**Table 8:** Sperm concentration and vitality of control and experimental groups

		**Sperm concentration **(million/g)	**Vitality** (eosin Test; %)
**Acute**	Control group	294 (205-338)	60 (57-70)
	Varenicline group	196 (143-225)^a^	22.5 (13-31)^a^
**Chronic**	Control group	209 (186-278)	77 (72-84)
	Varenicline group	123 (101-170)^b,c^	14.5 (6-20)^b,c^

^a^ significant decrease (*P<*0.05), vs acute control group, ^b ^significant decrease (*P<*0.05), vs chronic control group, ^c ^significant decrease (*P<*0.05), vs acute varenicline group

Values are expressed as the median (range)

## Discussion

Varenicline is a highly selective partial agonist for the nicotinic acetylcholine receptor a4b2 subtype, which is believed to be responsible for mediating the reinforcing properties of nicotine and more efficacious treatment; compare sustained-release bupropion or nicotine replacement therapy (15, 16). Varenicline has a proposed dual mechanism of relieving nicotine withdrawal symptoms (craving) and inhibiting rewarding effects during smoking (15, 17, 18). However, precautions should be taken with patients who have severe renal impairment. Acute renal failure due to varenicline use has been reported in the literature (19). In addition, an experimental study has indicated that treatment with varenicline for three days has nephrotoxic effects in rats (20). Recently, Singh *et al*. (5) reported that varenicline treatment to aid smoking cessation is associated with serious adverse cardiovascular events. *In vivo* studies also reported that the use of nicotine replacement therapies, varenicline, and bupropion, can cause endocrine changes that are consistent with impaired pancreatic β-cell function (21)

In the current study, we demonstrated that chronic varenicline treatment induced an increase in TBARS levels, which is an important sign of oxidative stress through increases in lipid peroxidation in the renal tissue (22). Furthermore, acute varenicline treatment caused a significant decrease in the levels of SOD in the renal tissue, while chronic varenicline treatment caused significant decrease in the levels of SOD, CAT, GPx, and GSH in the renal tissue. Oxidative stress is a condition associated with an imbalance between TBARS and the antioxidant defense system (23). Various experimental studies suggested that nephrotoxic drugs may also change the levels of TBARS, GPx, CAT, SOD, and GSH, which are commonly used to monitor the development and extent of renal tubular damage due to oxidative stress (22-25). Likewise, our results are in accordance with the oxidative stress studies. MPO, which is implicated in the pathogenesis of various inflammatory diseases, is a well-known inflammatory marker (26, 27). Previous studies indicated that increasing the MPO level reflected inflammatory conditions in renal injury (26-28). We observed that chronic varenicline treatment caused a significant increase in the levels of MPO in the renal tissue. Thus, it was indicated that chronic varenicline exposure might cause inflammation in the renal tissue. However, the biochemical analysis of the present study indicated that BUN, Cr, and testosterone levels in the serum of the rats in the acute and chronic varenicline treatment groups were not significantly affected.

The varenicline treated rats exhibited considerable cortical damage, tubular epithelial alterations, desquamated epithelial cells, and damaged glomerular structure in their kidneys. Furthermore, severe degenerative changes in the seminiferous tubules, significant loss in the number of germ cells in this seminiferous tubules, tubular atrophy, and tubular degeneration were observed in their testes. Our study’s histopathological results were in accordance with the biochemical data following varenicline exposure and led to the hypothesis that free radicals may have a critical role in the associated injury. 

Caspase 3, one of the 14 known members of the caspase family, is a key protease activated in the early stages of apoptosis (29). Apoptotic cells in testicular and renal tissues were marked using caspase 3 activity in our study. Apoptosis is a physiological process of selected cell deletion. As an antagonist of cell proliferation, apoptosis contributes to keeping the cell numbers in testicular and kidney tissues and helps to remove superfluous and damaged cells, but excessive apoptosis could cause destruction of male reproductive function and kidneys (30,31). In the present study, varenicline induced significant increase in caspase-3 expression indicating that varenicline provokes apoptosis in rat testes and kidneys.

In the present study, acute and chronic varenicline treatment caused significant changes in sperm characteristics (decreases of motility and concentration, and an increase of the abnormal sperm rate) compared with the control group. Similarly, both of the applications caused significant reductions in the right and left of epididymal weight as compared with the control groups. Based on the results of the current study, oxidative and histological damage in testicular tissue may be responsible for the toxicity and deteriorated sperm characteristics observed, and varenicline treatment may lead to infertility in rats. 

## Conclusion

The present study demonstrated the toxic effects of varenicline on kidney and testis tissues for the first time, to the best of our knowledge. Acute and chronic varenicline treatment caused a reduction in fertility and sperm production capacity in male rats, as indicated by altered biochemical, histological, and spermatological changes. Our results showed that male patients using varenicline due to smoking addiction should be closely followed both in terms of reproductive health and renal function. Additionally, further clinical investigations are needed to understand the underlying mechanism(s) of this status. 
